# Supplementary material for: Continued Vaccine Breakthrough Cases of Serotype 3 Complicated Pneumonia in Vaccinated Children, Portugal (2016–2019)
Source: Microbiol Spectr. 2022 Jul 6;10(4):e01077-22. doi: 10.1128/spectrum.01077-22 (PMC9431508; doi:10.1128/spectrum.01077-22)
Supplement: Supplemental file 1 — Supplemental material. Download spectrum.01077-22-s0001.pdf, PDF file, 0.1 MB [file spectrum.01077-22-s0001.pdf]

Supplemental Material

Continued vaccine breakthrough cases of serotype 3 complicated pneumonia in vaccinated children, Portugal (2016-19).

Catarina Silva-Costa†, Joana Gomes-Silva†, Marcos D. Pinho, Ana Friães, Mário Ramirez; José Melo-Cristino, on behalf of the Portuguese Group for the Study of Streptococcal Infections and the Portuguese Study Group of Invasive Pneumococcal Disease of the Pediatric Infectious Disease Society.

Instituto de Microbiologia, Instituto de Medicina Molecular, Faculdade de Medicina, Universidade de Lisboa, Lisboa, Portugal

†These authors have contributed equally to this paper

Table S1. Age group distribution of the 98 pediatric case-patients with *Streptococcus pneumoniae* infection included in this study, Portugal, 2010-2019

	Year			
Age group	2016	2017	2018*	2019
[0,1]	3	2	2	7
[2,5]	18	16	12	18
[6,17]	5	4	7	3

* The age of one patient in 2018 was unknown

§

Table S2. Serotype distribution among the 207 pediatric case-patients with *Streptococcus pneumoniae* infection, Portugal, 2010-2019

	Year*									
Serotype	2010	2011	2012	2013	2014	2015	2016	2017	2018	2019
1	7	5	4	2	1	2	1	1		
3	7	4	5	13	3	8	17	19	14	16
5		2								
6B			1				1			
6					1					
7F/7A	1	1	2	1	1					
8						1	1			4
9N										1
9V/9A	1		1							
10A			1							
11A/11D							1			
14	2					1	2		1	2
15A/15F					1					1
16F	1	1								
19A	2	2	1	1		1	1		2	
19F	1									
22F/22A								1		
23F		1								
33F/33A/37						1				
Not identified	7	2	4	1	3	1	2	1	5	4

* Data from 2010-2015 was presented previously [4]
